# Emphasizing the understanding of social determinants of health during clerkship

**DOI:** 10.1002/pdi3.100

**Published:** 2024-06-27

**Authors:** Shabih Manzar

**Affiliations:** ^1^ Louisiana State University Health Sciences Center at Shreveport Shreveport Louisiana USA

**Keywords:** clerkship, Pediatrics, social determinants of health

## 1 INTRODUCTION

**FIGURE 1:**
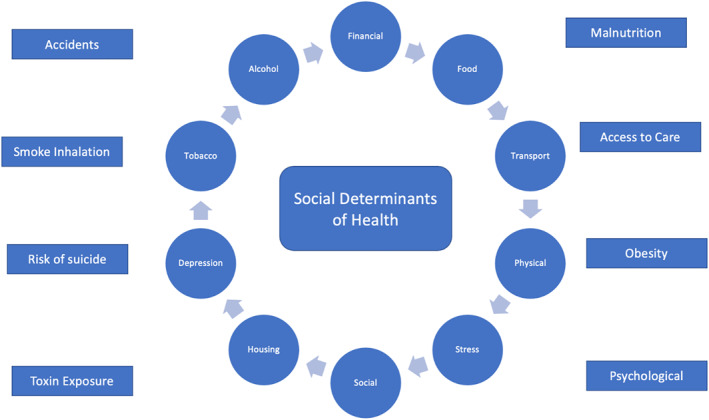
Social determinants of health and factors influencing them. Inner circles represent the social determinants of health (SDOH), while outer rectangles represent the diseases/disorders/health concerns and conditions associated with the SDOH.

Healthcare is affected by the social determinants of health (SDOH); therefore, it is essential to incorporate the understating of SDOH during medical training. SDOH are the non‐medical factors that influence health outcomes.^1^ SDOH include the conditions in which people are born, grow, work, live, and age. The essential SDOH are financial condition, food insecurity, transport facility, physical activity, stress, social connections, housing stability, depression, tobacco, and alcohol use (Figure 1).^2^, ^3^, ^4^, ^5^ Recently, Shah et al.^6^ successfully used an explicitly integrated social accountability principles rubric in family medicine clerkship during the 10‐min presentations and showed the value of looking beyond disease and focusing on SDOH. We conducted this study with the aim of addressing SDOH issues during the journal club (JC) sessions of pediatric clerkship.

## 2 METHODS

JC sessions are an hour session where students are provided with an article and have to complete a 5‐item questionnaire and submit it to the mentor (Appendix A). The students have to declare the purpose and design of the studies (Items 1 and 2, Appendix A) and note any flaw or limitation (Item 4, Appendix A). For example, the limitations in study one, suggested by some students, are incentives given to the participants and variation in text responses. Similarly, the limitations in study two, suggested by some students, are that there is no comparison group with different SDOH and possible subjectivity in financial coaching. In addition to these questionnaire responses, a live interactive discussion facilitates understanding the topic in detail. Twenty‐seven students rotating through the clerkship participated in the JC. One of the clerkship directors selected JC articles, including various pediatric issues. The JC article was provided to them a week prior to the JC session. For this study, the JC articles selected mainly focused on the social aspect of pediatric healthcare. The first article discussed the role of modest financial incentives in improving epinephrine autoinjector carriage rates among adolescents ages 15–19 with food allergies.^7^ The second study looked at the positive effects of medical‐financial partnership embedding financial coaching within pediatric primary care in improving low‐income families' adherence to recommended visits and vaccinations.^8^ The JC session was interactive, with active discussion among the students and the mentor. After reviewing the journal article, each student completed the 5‐item questionnaire (Appendix A) and submitted it to the clerkship director (paper's author). He then analyzed all responses, including the quantitative responses and qualitative statements (Appendix A).

## 3 RESULTS

The JC articles focused on the SDOH and a critical review demonstrated the students' understanding of integral association of SDOH with healthcare. The review of the responses to the 5‐item questionnaire showed understanding among students about SDOH. All students were able to address the purpose of the study. They delineated the study design was appropriate for both studies. Also, the JC participants thought that the statistics used in the study were appropriate. They all pointed out one drawback or limitation of the article, and all agreed with the conclusions.

## 4 DISCUSSION

The understanding of SDOH, as demonstrated by the group of medical students, was in agreement with the previous observation by Shah et al.^6^ The two articles reviewed specifically highlighted the pediatric aspects of SDOH.^7^, ^8^ The next step for the students is to have access to the SDOH screen on electronic healthcare records (EHR) when taking the history from the patients or parents. Many EHRs have recently incorporated SDOH in data acquisition (Appendix B, example from EPIC) in which students could access while accessing patients' records. By clicking the SDOH icon items on the EHR (Appendix B), the student would be provided with a questionnaire, and once answered by the patient, the response is recorded. If any specific determinant is concerning, it would be highlighted in red. Previously, we have demonstrated a variety of uses of SDOH in healthcare, including maternal health during pregnancy and the effects of maternal SDOH on neonatal birth weight.^2^, ^3^, ^4^, ^5^

In conclusion, as we observed and Shah et al.^6^ highlighted, we should incorporate the understanding of SDOH during clerkship. The fact that the students agreed with the conclusion does not guarantee they understood the papers completely. It is well possible that the conclusions of a paper do not reflect the results, as the aim of a JC is to critically review the paper, which does not mean that students need to agree with the content fully. Given these reservations, further studies are needed to confirm our findings.

## AUTHOR CONTRIBUTION

Shabih Manzar wrote the draft.

## CONFLICT OF INTEREST STATEMENT

The authors declare no conflicts of interest.

## ETHICS STATEMENT

Not applicable.

## CONSENT TO PARTICIPATE

Not applicable.

## Data Availability

Data sharing is not applicable to this article as no new data were created or analyzed in this study.

## APPENDIX A: JOURNAL CLUB ASSESSMENT SHEET

Student name:__________________________________________________________

Title of the article: _______________________________________________________

Answer the following questions:

(1) What was the purpose of the study?
(2) What study design did the authors use?
(3) Did the authors use the appropriate statistics (Table, Graph)
(4) Find ONE drawback or limitation of the article.
(5) Do you agree with the conclusions?

*Bring this sheet to the Journal Club assignment/discussion day and submit it to the mentor.*
